# Supplementary material for: Heterogeneity of glycan biomarker clusters as an indicator of recurrence in pancreatic cancer
Source: Front Oncol. 2023 Apr 12;13:1135405. doi: 10.3389/fonc.2023.1135405 (PMC10130372; doi:10.3389/fonc.2023.1135405)
Supplement: Supplementary file 1 [file DataSheet_1.pdf]

Supplementary Material

Heterogeneity of Glycan Biomarker Clusters as an Indicator of Recurrence in Pancreatic Cancer

Brian B. Haab*, Luke Wisniewski, Zachary Klamer, Samuel Braak, ChongFeng Gao, Chanjuan Shi, Peter Allen

*** Correspondence:** Brian B. Haab: brian.haab@vai.org

1 Supplementary Tables

1.1 Supplementary Table 1. Patient Data

ID	Gender	Age	Treatments	Group	Outcome
15-96	M	74	Gemcitabine/abraxane then FOLFOX	Recurrence	Recurrence at 1.5 years; OS 2.5 years
16-496	M	73	Single agent Gemcitabine then Abraxane	Recurrence	OS < 1 year
17-213	M	66	Gemcitabine and Abraxane	Recurrence	OS < 1 year
18-137	F	72	Gemcitabine and Xeloda; stopped Xeloda, initiated Gemcitabine and Abraxane; FOLFOX	Recurrence	Recurrence < 1 year; OS ~2 years
18-371	F	55	Gemcitabine and Capecitabine; FOLFIRINOX; Gemcitabine and Abraxane	Recurrence	Recurrence and progression < 1 year
18-760	F	63	Capecitabine/radiation then FOLFIRINOX then Gemcitibine/Abraxane	Recurrence	Recurrence at 1.5 years; mets at 2 years
19-167	F	41	FOLFIRINOX then Gemcitibine/Abraxane	Recurrence	Progression at < 1 year; OS < 2 years
20-272	F	80	Gemcitabine and Xeloda	Recurrence	OS < 1 year
15-658	M	39	FOLFIRINOX then Gemcitabine; Gemcitabine/nap-paclitaxel plus trial drug	Recurrence	Recurrence 3.5 years after surgery; OS 5.5 years
16-250	F	66	Capecitabine/radiation then Gemcitabine	Recurrence	Recurrence at 5 years; OS 6 years
17-543	F	80	Gemcitabine; added Cisplatin; radiation with oral capecitabine; Olaparib	Recurrence	Recurrence at ~2 years; OS 4.5 years
19-451	F	77	FOLFIRINOX then Gemcitabine/Abraxane	Recurrence	Recurrence at 1 year; progression at 2 years; stable at 3 years
20-333	M	62	FOLFIRINOX	Recurrence	Recurrence at ~ 2 years

14-767	M	73	Gemcitabine	No Recurrence	NED at 7.5 years
16-570	F	65	Capecitabine/radiation then Gemcitabine	No Recurrence	NED at 6 years
16-763	F	64	Gemcitabine	No Recurrence	NED at 6 years
18-460	M	69	FOLFIRINOX then radiation/Xeloda	No Recurrence	NED at almost 4 years
19-115	F	70	FOLFIRINOX	No Recurrence	NED at 3 years
19-296	M	65	FOLFIRINOX	No Recurrence	NED at 3 years
19-637	F	72	FOLFIRINOX	No Recurrence	NED at almost 3 years
20-281	M	68	FOLFIRINOX	No Recurrence	NED at almost 2 years
20-282	F	66	FOLFIRINOX	NA	Insufficient follow up

1.2 Supplementary Table 2. Receiver-operator characteristic analysis to determine cutoffs

See separate Excel file.

2 Supplementary Figures

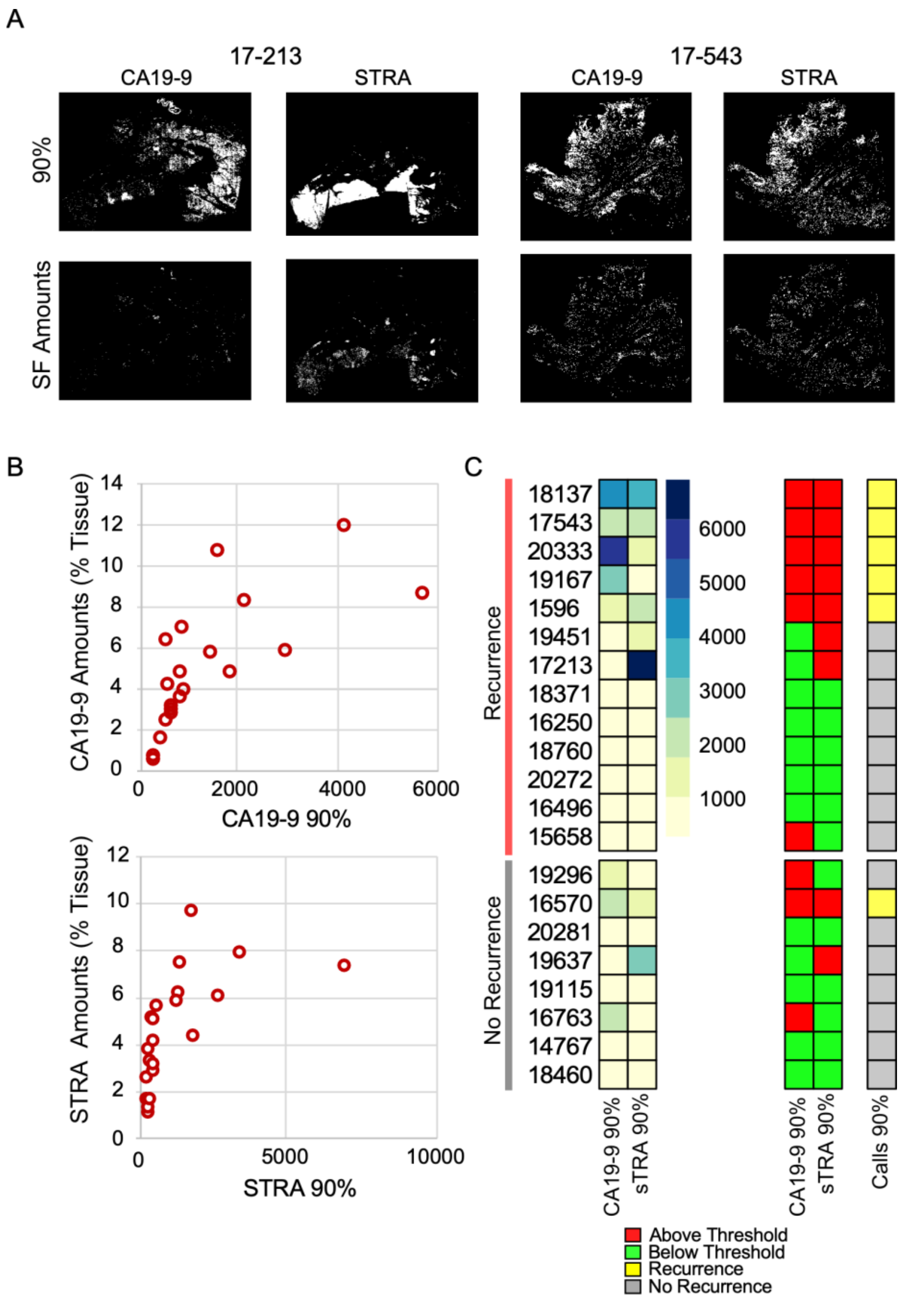

Supplementary Figure 1. Intensity-Based Thresholding. (A) Comparison of detected signal locations. The top panels show the top 10% of pixels based on intensity, and the bottom panels show the pixels found by SignalFinder. The intensity-based threshold was less selective. (B) Correlation of the two methods of quantification, showing general correlation at lower intensities but much less at higher intensities. (C) Classification using intensity-based thresholds, based on the same system as described in the main text. The accuracy was not statistically significant.

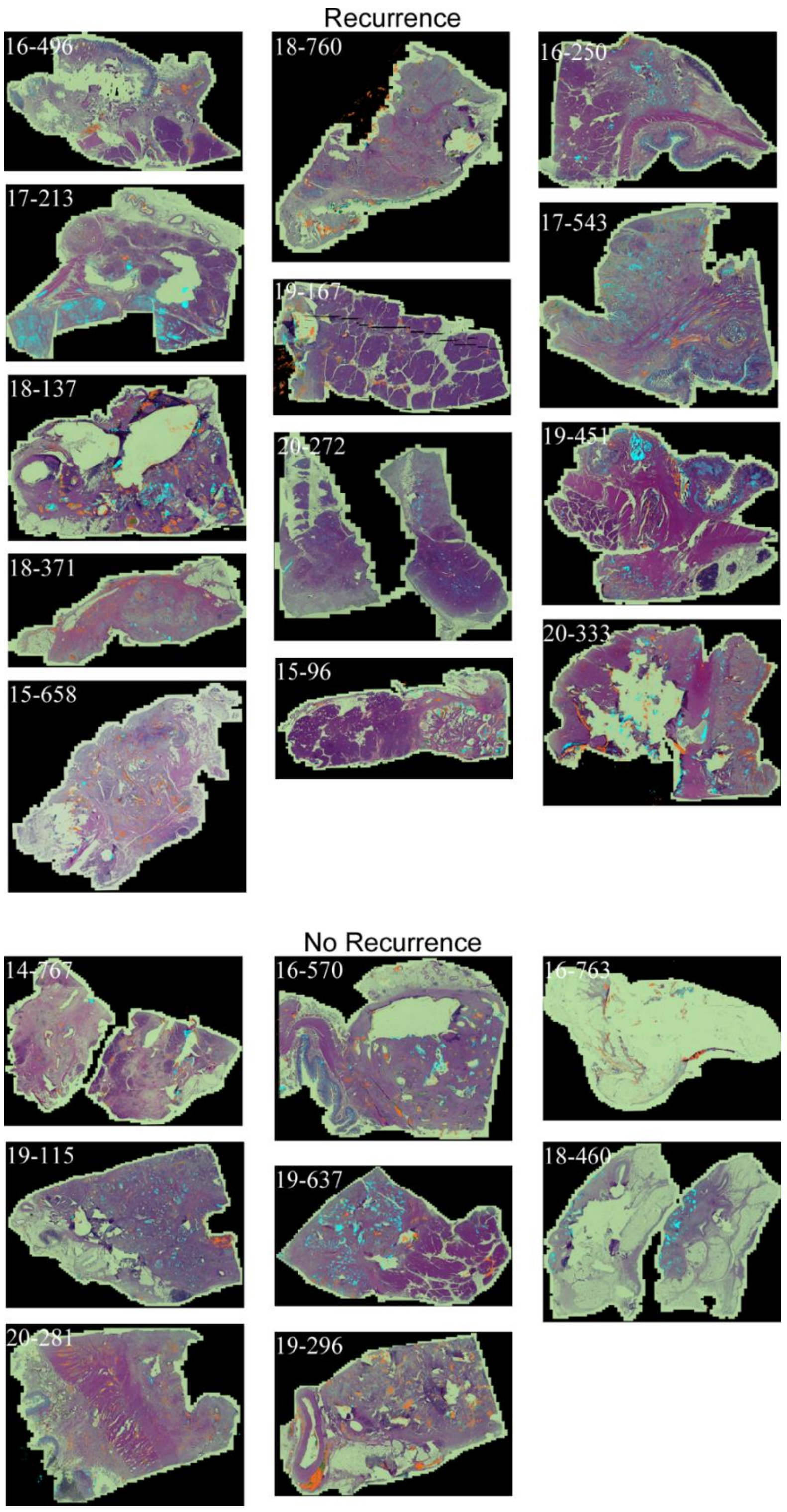

Supplementary Figure 2. Whole-block tumor specimens. The images show the H&E-stained tissue overlaid with the SignalFinder-detected signals from STRA (cyan) and CA19-9 (orange).

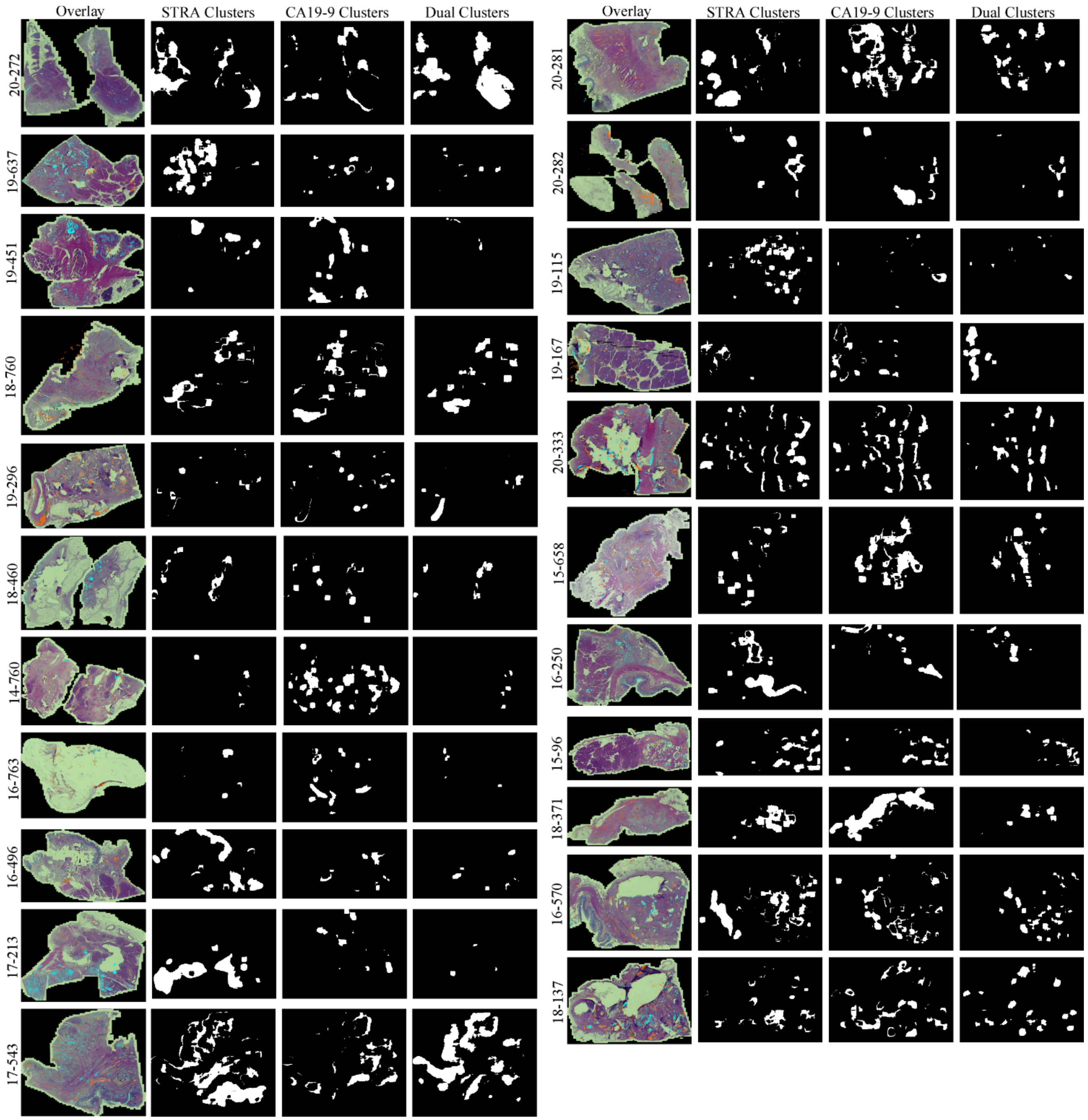

Supplementary Figure 3. Cluster maps. Each row shows the signal-overlaid H&E image followed by the maps of the STRA-only, CA199-only, and dual clusters.

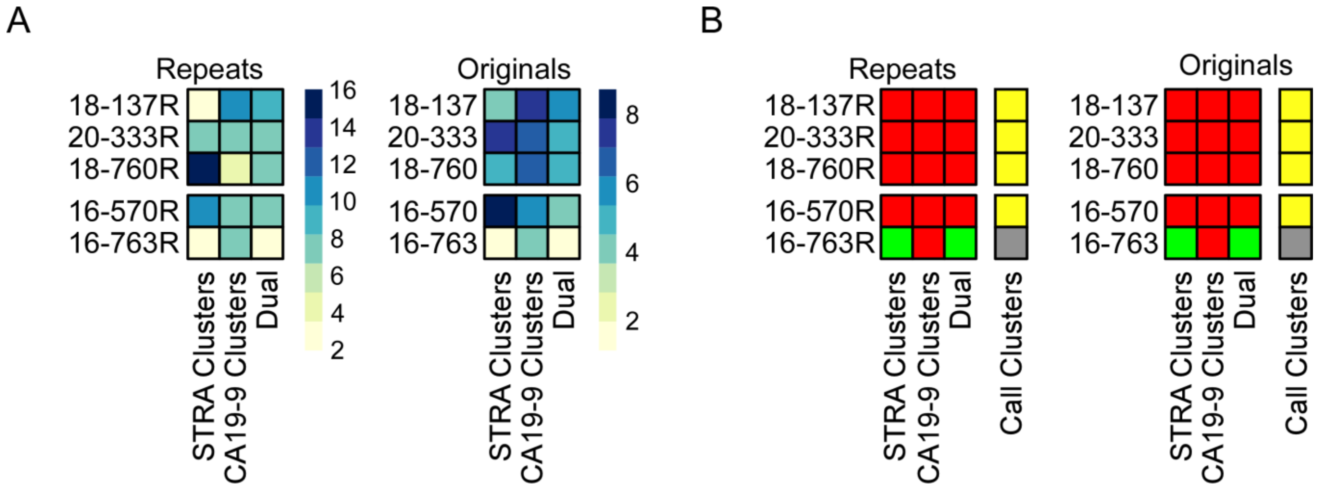

Supplementary Figure 4. Test of consistency between sections. (A) The matrix shows the quantifications of the three types of clusters in the original sections and the repeat sections, taken 10-20 mm removed from the original sections. (B) Thresholded data using the same thresholds and classification rule for both sets.
